# Phytochemical Screening and Acute Oral Toxicity Study of Java Tea Leaf Extracts

**DOI:** 10.1155/2015/742420

**Published:** 2015-12-27

**Authors:** Raghunath Pariyani, Intan Safinar Ismail, Amalina Ahmad Azam, Faridah Abas, Khozirah Shaari, Mohd Roslan Sulaiman

**Affiliations:** ^1^Laboratory of Natural Products, Institute of Bioscience, Universiti Putra Malaysia, 43400 Serdang, Selangor, Malaysia; ^2^Department of Chemistry, Faculty of Science, Universiti Putra Malaysia, 43400 Serdang, Selangor, Malaysia; ^3^Department of Food Technology, Faculty of Food Science and Technology, Universiti Putra Malaysia, 43400 Serdang, Selangor, Malaysia; ^4^Department of Biomedical Sciences, Faculty of Medicine and Health Sciences, Universiti Putra Malaysia, 43400 Serdang, Selangor, Malaysia

## Abstract

The term Java tea refers to the decoction of* Orthosiphon stamineus *(OS) Benth (Lamiaceae) leaves, which are widely consumed by the people in Europe and South East Asian countries. The OS leaves are known for their use in traditional medicinal systems as a prophylactic and curative agent for urinary stone, diabetes, and hypertension and also as a diuretic agent. The present study was aimed at evaluating its possible toxicity. Herein, the major phytochemical constituents of microwave dried OS leaf, which is the common drying process for tea sachets in the market, were also identified. The acute oral toxicity test of aqueous, 50% aqueous ethanolic, and ethanolic extracts of OS was performed at a dose of 5000 mg/Kg body weight of Sprague-Dawley rats. During the 14-day study, the animals were observed for any mortality, behavioral, motor-neuronal abnormalities, body weight, and feed-water consumption pattern. The hematological and serum biochemical parameters to assess the kidney and liver functions were carried out, along with the histological analysis of these organs. It was found that all microwave dried OS leaf extracts did not cause any toxic effects or mortality at the administered dose. No abnormality was noticed in all selected parameters in rats of both sexes as compared with their respective control groups. Thus, the possible oral lethal dose for microwave dried Java tea leaves is more than 5000 mg/Kg body weight.

## 1. Introduction

Nowadays, the usage of products of natural origin such as those derived from plant, animal, or marine sources as health supplements, revitalizers, and agents in disease prevention is in rise. The concept of food as medicine has evolved and been in practice a long time ago. Likewise, natural product remedies were the most popular and reliable method among traditional people in the disease prevention and treatment, as evident from ancient literature. It is truly universal as could be seen in different regions of the world using the approach of natural product therapies, but under different names which are mainly the Traditional Chinese Medicine (TCM), Ayurveda, Siddha, and Unani. Even though the names are different, they all rely on a general platform of using materials from nature in the cure and protection against diseases.

As a continuum, more scientific research was directed at identification and isolation of bioactive principles from natural sources. Despite the development of successful drugs from natural products, most of the synthesized drugs could be coupled with distress caused by the side effects. This has catalyzed the exploration and usage of natural resources for their medicinal properties. Among the natural sources, medicinal plants are the main contributing class.

The wide market and growing demand for natural medicinal plants in the forms of food or health supplement, health drink or tonic, and many other forms have fuelled the development and introduction of multiple forms of a single product derived from the particular plant material with added claims such as utilizing advanced processing technologies and products enriched with certain bioactive components. The different process variables employed in the development of such products resulted in qualitative or quantitative changes in the chemical profiles of the plant material causing the alteration of the biological profile [1, 2]. Most of the researches focus on optimizing and advancing the extraction methods so as to extract maximum biological active material. However, assessing the toxicity profile of such products is of vital importance [3]. It becomes particularly essential since most people have a general belief that all natural products are safe and free to access and consume since most of these products are marketed as supplements [4]. Hence it is imperative to have proper chemical, toxicological, and safety data for the usage of plants with traditional claims on health benefits.

*Orthosiphon stamineus* Benth (OS) of the family Lamiaceae is one of such plants. The products of OS are well marketed through pharmacies and supermarkets in Europe and South East Asian countries. The decoction of OS, which is popularly known as* Java tea*, is believed to improve health and also used in the treatment of kidney or bladder inflammation, gout, and diabetes [5–8]. A variety of Java tea products differing in processing methods are available in the market, among them the main variable being the drying method of leaves. Products employing microwave drying method is on the rise as it is one of the fast and easiest methods of drying, especially in the industrial scale production [9]. However, the chemical constituent profile and toxicity data of such product are not available yet. An acute toxicity study is a preliminary, yet an important study which could shed light on the general safety of a substance. Generally, a single high dose of the material is administered to animals and they are observed for behavioral, motor-neuronal changes and mortality [10]. The median lethal dose (LD_50_) could be calculated so as to decide the safety window for usage. This present study is aimed at evaluating the acute oral toxicity of commonly used microwave dried OS leaf and also at identifying its major phytochemical constituents.

## 2. Materials and Methods

### 2.1. Plant Collection and Extraction

Fresh leaves of 2-month-old OS plants grown under same environmental and growth conditions were collected from Taiping, Perak, Malaysia, in January 2012. The plant sample was authenticated by the botanist at Unit of Biodiversity, Institute of Bioscience, Universiti Putra Malaysia, and a voucher specimen (SK1997/12) was deposited at the unit herbarium. The leaves were washed in running water, padded with tissue paper, and then dried using industrial scale continuous drying microwave equipment with an output frequency of 2.45 ± 0.05 GHz for a period of 40 s. The selection of the microwave frequency was based on a trial and error basis in ensuring an efficient drying process that caused little stress to the composition of the leaves as assessed by their color and texture. The dried leaf material was then ground in a blender to powder; size uniformity was ensured by sieving through a stainless steel mesh of 200 mm diameter and stored in airtight containers at 3 ± 2°C for further processing. The OS leaf powder was then extracted with water, 50% aqueous ethanol and ethanol to yield aqueous (OSA), 50% aqueous ethanolic (OSF), and ethanolic (OSE) extracts, by ultrasonic assisted extraction method. Weighed quantity of the leaf material was extracted in measured volume of solvent (ratio of 1 g : 20 mL) by subjecting to sonication for 30 min while maintaining the sonication bath at a temperature window of 30–40°C. The extract was filtered before repeating twice with fresh solvent and sonication step, for each time. The filtrate was combined and the solvent was removed using rotary evaporator at 40°C. The resulting crude extract was kept frozen until used for the study.

### 2.2. Phytochemical Characterization of the OS Extracts

The LC-MS/MS analysis was carried out using an AB Sciex 3200 QTrap with Perkin Elmer UHPLC FX15. The negative ion mass spectra were obtained using a scan range of 100–1200 *m*/*z* for full scan, 50–1200 *m*/*z* for MS/MS scan. A Zorbax C18 column of 150 mm × 4.6 mm × 5 *μ*m was used in obtaining the separation of the phytoconstituents. Solvents A and B were water and acetonitrile, with 0.1% formic acid and 5 mM ammonium formate, respectively. The HPLC profile was acquired using a gradient run method programmed as 10% B to 90% B from 0.01 min to 8 min, before holding at 90% B for 2 min. The gradient program was then changed from 90% B to 10% B in 0.1 min for reequilibration over a period of 5 min before the next injection. The processing and identification of the peaks were done using ACD/MS workbook suite and also comparing with the literature data [11, 12].

### 2.3. Experimental Animals

Eight-week-old Sprague-Dawley rats of both sexes, weighing 140–160 g, were purchased from Animal Resource Unit, Faculty of Medicine and Health Sciences, Universiti Putra Malaysia (Serdang, Malaysia). All rats were acclimatized to the laboratory condition for a period of one week prior to dosing. The rats were housed in air conditioned room at 24 ± 2°C on a 12/12 h light-dark cycle. Three rats were housed per polycarbonate cage and had free access to rodent chow (Specialty Feeds, Glen Forrest, Western Australia) and water* ad libitum*. All the animal experimental protocols were approved (approval number: UPM/FPSK/PADS/BR-UUH/00460) by Animal Care and Use Committee (ACUC), Faculty of Medicine and Health Sciences, Universiti Putra Malaysia. All efforts were made to minimize suffering and distress of rats.

### 2.4. Acute Toxicity Study

The rats were divided into 4 groups, with 12 rats (6 males and 6 females) per group. Group 1 served as the control, and the other three groups were treated with OSA, OSF, and OSE. The control group was given 5% Tween-20, while the treatment groups received OS leaf extract at 5000 mg/Kg body weight (BW) suspended in 5% Tween-20 through oral gavage. A 5000 mg/Kg BW dose of OS was chosen for the limit test in accordance with The Organization for Economic Cooperation and Development (OECD) guidelines for the Testing of Chemicals [13]. The administered volume was adjusted to 8 mL/Kg BW for every rat. The vehicle and the OS extract were administered only once (on day 0) at the start of the experiment, and the rats were monitored for 14 days.

#### 2.4.1. Cage Side Observation and Body Weight

The general behavior, body weight, and feed-water intake of the rats were observed during the acclimatization period. After administration of the OS leaf extract, each rat was continuously monitored for the first hour followed by every 2 hours till 12 h and then every day for 14 days. The monitored parameters included properties of skin and fur, eyes, respiratory pattern, autonomic nervous system features such as salivation, diarrhea, and urination, central nervous system features such as tremors, ptosis, relaxation, changes in the level of activity, gait, and posture, and any other abnormal behavior [14]. The feed and water intake pattern of the animals was observed throughout the study period. The weight of the animal was recorded on alternate days and the individual record on all observations was maintained for each rat.

#### 2.4.2. Hematological Analysis

On the 14th day of the study, the animals were fasted overnight but were allowed water* ad libitum*. The blood drawn by cardiac puncture method was collected into plain tubes and Vacutainer coated with EDTA. The blood sample collected to EDTA coated tube was used in analysis of hematological parameters, such as total red blood cells (RBC), total white blood cells (WBC), hemoglobin (Hb), hematocrit (HCT), mean corpuscular hemoglobin (MCH), mean corpuscular hemoglobin concentration (MCHC), lymphocytes, platelet count, red blood cell distribution width (RDW), platelet distribution width (PDW), mean platelet volume (MPV), and platelet larger cell ratio (P-LCR), which were estimated using automated hematology analyzer.

#### 2.4.3. Biochemical Analysis

The blood sample collected into the plain tubes was left at 4°C for 3 h and then was centrifuged at 3000 rpm for 10 minutes to separate the serum. The concentrations of biochemical markers such as creatinine, urea, glucose, aspartate aminotransferase (AST), alanine aminotransferase (ALT), and bilirubin were estimated using standard assay kits.

#### 2.4.4. Histopathology

The rats were painlessly killed under ether anesthesia and the organs (liver, kidney, stomach, and testis/ovary) were harvested for histopathological examination. The weight of the organs was recorded after being washed with normal saline and dried using blotting paper. The relative weight index of each organ to its body weight was calculated by the formula: (weight of organ/bodyweight of rats on the day of sacrifice) × 100% [15].

The organs were then fixed in 10% formalin. The fixed tissues were embedded and cut into 5 *μ*m thick sections. The hematoxylin and eosin stained sections were observed under light microscope.

### 2.5. Statistical Analysis

One-way analysis of variance (ANOVA) was performed using GraphPad Prism V 6.0 (GraphPad Software Inc., San Diego, CA, USA); Dunnett comparison test was chosen as* post hoc* analysis method where *p* ≤ 0.05 was considered to be statistically significant. The results were expressed as mean ± SEM.

## 3. Results

### 3.1. Phytochemical Characterization of the Microwave Dried OS Extracts

**Figure 1:**
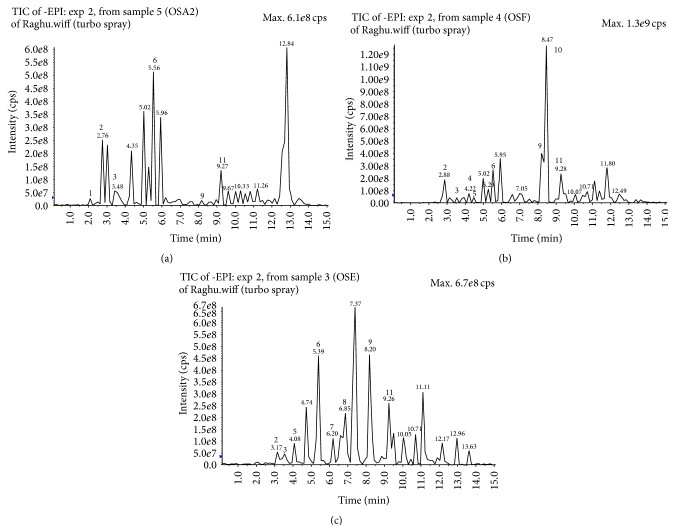
LC-MS/MS chromatogram of (a) aqueous, (b) 50% aqueous ethanolic, and (c) ethanolic extracts of OS leaves.

The LCMS/MS analysis of the aqueous, 50% ethanolic, and ethanoic extracts (Figure 1) exhibited quite a difference among them with the presence of rosmarinic acid, protocatechuic acid, caffeic acid, tetramethoxy chalcone derivatives, ferulic acid, syringic acid, kaempferol methyl ether, succinic acid, protocatechuic acid hexoside, and Luteolin as identified and listed in Table 1.

Rosmarinic acid, a marker constituent of OS, was identified in all the analyzed extracts. However, two other bioactive constituents, namely, Luteolin and ferulic acid, were present in the ethanolic extract only. Likewise, succinic acid and tetramethoxy chalcone were detected only in aqueous and 50% aqueous ethanolic extracts, respectively.

### 3.2. Acute Toxicity Study

#### 3.2.1. Cage Side Observation

All the treatment groups rats recorded normal behavioral, motor, and neuronal functions for all the administered OS extracts with no mortality observed. The monitoring of skin and fur, eyes, behavioral pattern such as gait and posture, and autonomic and central nervous system activities of treatment rats remained unchanged with the treatment of OS when compared with those of control group. This showed that the oral LD_50_ of aqueous, 50% aqueous ethanolic, and ethanolic extracts of OS leaves was greater than 5000 mg/Kg body weight.

#### 3.2.2. Body Weight Measurement

**Figure 2:**
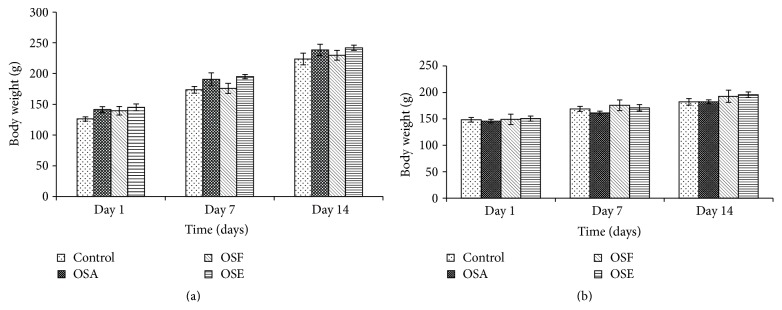
Effect of OS extracts on body weight in male (a) and female (b) rats (6 males and 6 females) during 14-day oral acute toxicity study. Data are expressed as mean ± standard error of the mean (SEM).

**Figure 3:**
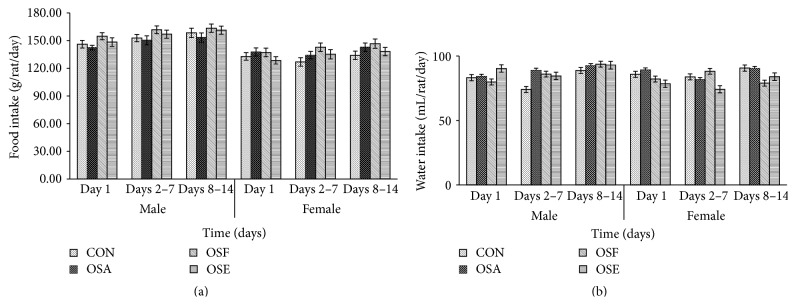
Effect of OS extracts on food (a) and water (b) intake in male and female rats (6 males and 6 females) during 14-day oral acute toxicity study. Data are expressed as mean ± standard error of the mean (SEM) and analyzed by one-way ANOVA, followed by Dunnett's test (*p* < 0.05) as compared to that of control groups (CON).

The body weight of the treatment group rats of both sexes did not record any statistically significant (*p* > 0.05) changes when compared with the control, throughout the study period (Figures 2(a) and 2(b)). The feed and water consumption pattern of the rats was regular and consistent throughout the experimental period (Figures 3(a) and 3(b)).

#### 3.2.3. Hematological Analysis

The effects of a single oral administration of 5000 mg/Kg body weight dose of OSA, OSF, and OSE on hematological parameters of rats of both sexes are shown in Table 2. The analyzed parameters did not record any statistically significant (*p* > 0.05) changes as compared to respective control groups.

#### 3.2.4. Biochemical Analysis

The effects of a single oral administration of 5000 mg/Kg body weight dose of OSA, OSF, and OSE on biochemical parameters of rats of both sexes are shown in Table 3. The measured parameters recorded statistically insignificant (*p* > 0.05) changes as compared to respective control groups.

#### 3.2.5. Histopathology and Relative Organ Weight

The microscopic evaluation of the tissue sections of either sex of the OS treated rats did not show any lesions or abnormal histopathological changes as compared to their respective control groups (data not shown). The organs retained normal texture and appearance on gross examination. The relative organ weight index of the rats of either sex treated with OS extracts did not show significant changes as compared to their respective control groups (Table 4).

## 4. Discussion

Natural product remedies are popular and gaining wide acceptability among people in the prevention and cure of diseases. The general belief that the products of natural origin are safe and free from serious side effects which the synthetic drugs possess drives people towards using the natural medicines. However, many of the natural product formulations available in the market do not have sufficient scientific data on their safety and toxicological profile. This assumes particular importance since the natural products are more often used under self-medication without a medical supervision. Hence, proper scientific knowledge on the toxicity and safe administration level of the natural medicines is crucial.

The present study focused on the preliminary phytochemical and acute toxicity evaluation of the microwave dried* Orthosiphon stamineus* (OS) or* Java tea* leaves, a traditional medicinal plant. The aqueous extract was chosen for the study as it represented the most common mode of consumption of OS in the form of decoction. The selection of 50% ethanol and ethanol extracts was guided by the literature reports on their efficacy in elucidating diuretic, antidiabetic, and nephrolithiasis effects [16–18]. The 14-day acute toxicity study of the said solvent extracts of OS did not cause any mortality or behavioral, motor-neuronal abnormalities in rats. Mohamed et al. [19] and Abdullah et al. [20] assessed the toxicity profile of shade dried leaves of OS and also found that OS did not show toxicological signs on rats. The findings from the current study established the safety of microwave dried* Java tea *leaves.

The monitoring of body weight and feed/water consumption of the experimental animals is important while studying the toxicity and safety of a natural product since it hints at the physiological and metabolic status of the animals and gets rid of the researcher from deriving any “false” observations due to nutritional abnormalities of the rats. In the current study, the weight gain shown by all the rats was comparable and followed a general trend. None of the experimental groups suffered loss in weight or gained overweight which suggested that the OS extracts did not induce significant changes in the appetite and did not exert any deleterious effects on the general health status and metabolic growth of the rats. It was also noted that the feed and water intake of the rats from the time of acclimatization till the end of the experiment followed a common and steady pattern. The pattern of body weight and feed consumption was not altered significantly (*p* > 0.05) by the administration of aqueous, 50% aqueous ethanolic, and ethanolic extracts of OS which suggested that the plant extracts did not induce any deleterious effects on growth and development of the rats.

The relative organ weight index is used as another basic indicator to assess the deleterious effects of the plant metabolites. The effect of toxic substances on the internal organs could be identified by assessing the relative organ weight as the index gives a preliminary insight to the swelling or damage caused by any harmful agent [21]. In this study, the relative organ weight of liver, kidneys, stomach, testis, or ovary was determined and none of the treated male and female rats showed significantly different (*p* > 0.05) relative organ weight as compared with their respective control groups.

The assessment on the hematological parameters is important as it can point directly to the systemic effects caused by the administered extract. From the results, the hematological profile of all the treated rats showed no significant difference (*p* > 0.05) in comparison with the control group.

Several important biochemical parameters were also included in this toxicity study. The primary organs prone to the toxic effects of medicines are the kidney and liver. The kidney function parameters such as serum creatinine, urea, and total protein were determined, while the level of AST and ALT was determined to assess the liver function. The results of the experiment suggested that the kidney and liver functions were not altered for both the treated male and female rats. There were no statistically significant differences in creatinine, urea, total protein, AST, and ALT levels between controls and treated animals. Hence, these findings suggest that either aqueous, 50% aqueous ethanolic, or ethanolic extracts of OS did cause any deleterious effects on kidney and liver of the rats.

Histopathological studies were conducted on kidney, liver, stomach, testis, and ovary of all the rats. Gross examination of the organs did not show any signs of necropsy and abnormal morphological changes. The microscopic examination of the hematoxylin eosin stained tissue sections also recorded insignificant changes as compared with the control rats' tissues.

The results from this acute oral toxicity study suggested that the aqueous, 50% ethanolic, and ethanolic extracts of microwave dried OS leaves are relatively nontoxic and the no-observed-adverse-effect level (NOAEL) of OS was determined as 5000 mg/Kg body weight/day. However, further toxicity assessment such as subacute, chronic, or genotoxic studies using repeated dose of Java tea should be conducted to confirm its safety on prolonged use.

## 5. Conclusion

The aqueous, 50% aqueous ethanolic, and ethanolic extracts of microwave dried OS leaves contain pharmacologically active compounds belonging to flavonoids and phenolics, which have documented high antioxidant and other beneficial pharmacologic activities. The microwave dried OS leaves did not exhibit obvious toxicity even at a high dose of 5000 mg/kg body weight. Thus the microwave dried OS leaves, which are generally consumed as* Java tea*, are presumed safe to be used as an oral health supplement. However, safety of repeated dose administration could be studied by subacute and chronic toxicity studies to further the understanding.

## Figures and Tables

**Table 1:** Retention times, MS, and MS/MS values of the major constituents present in OS leaves crude extracts identified via QTrap LCMS/MS with UHPLC system.

Peak	Retention time	Molecular ion peak (M-H)^−^	MS^2^ fragment ion intensity	Tentative compounds identified
1	2.1	117	73^*∗*^, 99.0, 69.7, 67.6, 71.5	Succinic acid
2	2.76	197	135.0^*∗*^, 123.0, 134.0, 179.0	Syringic acid
3	3.48	153	109^*∗*^, 65.0, 91.0, 108.0, 122.0	Protocatechuic acid
4	4.22	137	137^*∗*^, 108.0, 93.0, 65.0, 63.0	4-Hydroxy benzoic acid
5	4.46	174	135.0^*∗*^, 89.0, 108.0, 132.0	Caffeic acid
6	5.52	359.1	133^*∗*^, 123, 161.1, 179.1, 197.1	Rosmarinic acid
7	6.2	193.1	133.0^*∗*^, 137.0, 161.0	Ferulic acid
8	6.62	285	133^*∗*^, 65, 175.1, 199.0, 217.1	Luteolin
9	8.20	299.1	284.1^*∗*^, 133.0, 227.2, 256.2, 211.2	Kaempferol methyl ether
10	8.47	343.1	270.1^*∗*^, 170.3, 198.3, 214.3	Tetramethoxy chalcone derivative
11	9.27	316.3	315.3^*∗*^, 297.4, 197.2, 185.2, 169.2	Protocatechuic acid hexoside

^*∗*^Base peak.

**Table 2:** Effect of OS extracts on hematological parameters in rats during 14-day oral acute toxicity study.

Parameter	Unit	CON	OSA	OSF	OSE
Male					
WBC	10^9^/L	5.45 ± 0.54	6.12 ± 1.24	5.6 ± 0.52	6.28 ± 0.87
RBC	10^12^/L	5.81 ± 0.20	5.7 ± 0.16	6.03 ± 0.21	5.62 ± 0.29
PLT	10^9^/L	942.2 ± 83.22	865.5 ± 158.5	779.8 ± 215.8	866 ± 144.2
LYM	10^9^/L	4.05 ± 0.41	6.65 ± 1.09	4.25 ± 0.36	5.07 ± 0.69
Hb	g/dL	12.25 ± 0.37	12.15 ± 0.35	12.37 ± 0.27	12.65 ± 0.38
HCT	%	38.32 ± 1.18	37.6 ± 1.14	38.9 ± 0.84	37.38 ± 1.85
MCV	fL	66.03 ± 0.57	65.93 ± 0.44	64.68 ± 1.06	66.62 ± 0.88
MCH	pg	21.12 ± 0.11	21.3 ± 0.08	20.53 ± 0.20	22.28 ± 1.16
MCHC	g/dL	31.98 ± 0.20	32.3 ± 0.15	31.78 ± 0.38	33.5 ± 1.56
RDW	%	13.97 ± 0.49	13.45 ± 0.31	14.18 ± 0.66	14.1 ± 0.28
PDW	fL	8.14 ± 0.09	8.57 ± 0.25	9.22 ± 0.71	9.17 ± 0.42
MPV	fL	6.88 ± 0.10	7.08 ± 0.11	7.2 ± 0.28	7.48 ± 0.18
P-LCR	%	5.8 ± 0.50	6.95 ± 0.81	8.75 ± 2.32	9.82 ± 2.02
Female					
WBC	10^9^/L	5.13 ± 0.42	4.02 ± 0.19	6.25 ± 0.65	5.95 ± 1.0
RBC	10^12^/L	6.62 ± 0.13	6.51 ± 0.26	6.31 ± 0.12	6.44 ± 0.16
PLT	10^9^/L	843.7 ± 98.89	1018 ± 151.5	991 ± 181.4	942.2 ± 109.6
LYM	10^9^/L	4.12 ± 0.43	2.58 ± 0.37	4.97 ± 0.51	4.92 ± 0.85
Hb	g/dL	13.45 ± 0.18	13.55 ± 0.51	13.7 ± 0.2	13.35 ± 0.23
HCT	%	40.1 ± 0.65	40.57 ± 1.82	38.8 ± 0.77	39.88 ± 0.81
MCV	fL	60.58 ± 0.74	62.27 ± 0.81	61.55 ± 0.66	62.02 ± 0.66
MCH	pg	20.82 ± 0.30	20.82 ± 0.25	21.18 ± 0.12	21.32 ± 0.24
MCHC	g/dL	33.55 ± 0.13	33.47 ± 0.29	34.42 ± 0.42	34.38 ± 0.37
RDW	%	12.2 ± 0.31	12.2 ± 0.20	11.65 ± 0.41	11.4 ± 0.17
PDW	fL	8.43 ± 0.23	8.1 ± 0.14	8.37 ± 0.22	8.33 ± 0.17
MPV	fL	7.08 ± 0.12	6.79 ± 0.10	6.87 ± 0.13	6.95 ± 0.13
P-LCR	%	6.45 ± 0.49	5.42 ± 0.47	5.62 ± 0.54	5.87 ± 0.54

Data are expressed as mean ± standard error of the mean (SEM) and analyzed by one-way ANOVA, followed by Dunnett's test (*p* < 0.05) as compared to the respective parameter value of control groups (CON) (*6 males and 6 females*).

**Table 3:** Effect of OS extracts on serum biochemical parameters in rats during 14-day oral acute toxicity study.

Parameter	CON	OSA	OSF	OSE
Male				
Urea (mmol/L)	6.883 ± 0.25	5.533 ± 0.40	5.417 ± 0.27	6.417 ± 0.22
Creatinine (*μ*mol/L)	43.83 ± 1.01	42.83 ± 1.35	40.33 ± 1.62	42.67 ± 1.69
Glucose (mmol/L)	10.83 ± 0.72	9.318 ± 0.74	10.61 ± 0.91	10.18 ± 0.87
Total protein	52.52 ± 0.99	51.23 ± 1.41	50.08 ± 0.99	51.52 ± 0.93
ALT (U/L)	44.58 ± 4.11	47.52 ± 3.23	33.77 ± 3.96	39.18 ± 8.87
AST (U/L)	188.2 ± 32.25	157.3 ± 12.66	108.9 ± 12.98	112.9 ± 20.97
Bilirubin (mg/dL)	0.475 ± 0.13	0.45 ± 0.08	0.45 ± 0.05	0.36 ± 0.08
Female				
Urea (mmol/L)	6.633 ± 0.47	5.5 ± 0.29	6.1 ± 0.29	6.217 ± 0.38
Creatinine (*μ*mol/L)	45.5 ± 0.81	47.83 ± 1.62	44.67 ± 0.84	44.83 ± 0.98
Glucose (mmol/L)	8.877 ± 0.59	9.093 ± 1.06	6.225 ± 0.95	9.26 ± 0.60
Total protein	58.02 ± 2.04	59.05 ± 2.72	53.1 ± 3.98	57.45 ± 2.21
ALT (U/L)	40.58 ± 2.83	45.47 ± 3.76	31.67 ± 4.05	35.15 ± 5.47
AST (U/L)	121.3 ± 12.75	153.2 ± 12.06	95.73 ± 14.29	92 ± 11.35
Bilirubin (mg/dL)	0.5 ± 0.07	0.575 ± 0.09	0.55 ± 0.09	0.5 ± 0.09

Data are expressed as mean ± standard error of the mean (SEM) and analyzed by one-way ANOVA, followed by Dunnett's test (*p* < 0.05) as compared to respective parameter value of control groups (CON: *6 males and 6 females*).

**Table 4:** Effect of OS extracts on relative organ weights in rats during 14-day oral acute toxicity study.

	CON	OSA	OSF	OSE
Male				
Liver	4.83 ± 0.10	4.72 ± 0.19	4.83 ± 0.26	5.43 ± 0.19
Kidney (R)	0.77 ± 0.01	0.72 ± 0.01	0.73 ± 0.04	0.78 ± 0.04
Kidney (L)	0.75 ± 0.01	0.73 ± 0.01	0.74 ± 0.03	0.70 ± 0.02
Stomach	0.99 ± 0.03	0.91 ± 0.03	0.90 ± 0.02	1.02 ± 0.05
Testis	0.86 ± 0.02	0.84 ± 0.04	0.85 ± 0.04	0.84 ± 0.03
Female				
Liver	3.99 ± 0.16	4.59 ± 0.13	4.56 ± 0.28	4.75 ± 0.29
Kidney (R)	0.78 ± 0.01	0.80 ± 0.01	0.81 ± 0.03	0.83 ± 0.03
Kidney (L)	0.75 ± 0.01	0.75 ± 0.02	0.78 ± 0.02	0.80 ± 0.03
Stomach	1.05 ± 0.03	1.03 ± 0.03	0.96 ± 0.04	1.01 ± 0.09
Ovary	0.44 ± 0.01	0.53 ± 0.08	0.43 ± 0.01	0.45 ± 0.03

Data are expressed as mean ± standard error of the mean (SEM) and analyzed by one-way ANOVA, followed by Dunnett's test (*p* < 0.05) as compared to respective parameter value of control groups (CON: *6 males and 6 females*).
